# A chronic high‐fat diet does not exacerbate muscle atrophy in fast‐twitch skeletal muscle of aged mice

**DOI:** 10.1113/EP091106

**Published:** 2023-04-19

**Authors:** Tsutomu Tagawa, Hiroaki Eshima, Saori Kakehi, Ryuzo Kawamori, Hirotaka Watada, Yoshifumi Tamura

**Affiliations:** ^1^ Department of International Tourism Nagasaki International University Nagasaki Japan; ^2^ Department of Metabolism & Endocrinology Juntendo University Graduate School of Medicine Tokyo Japan; ^3^ Sportology Center Juntendo University Graduate School of Medicine Tokyo Japan; ^4^ Center for Therapeutic Innovations in Diabetes Juntendo University Graduate School of Medicine Tokyo Japan; ^5^ Center for Identification of Diabetic Therapeutic Targets Juntendo University Graduate School of Medicine Tokyo Japan

**Keywords:** ageing, cross‐sectional area, fast‐twitch muscle, high‐fat diet, myofibre diameter, skeletal muscle

## Abstract

Obesity and ageing reduce muscle mass and lead to deficits in muscle maintenance, but it is not known whether obesity accelerates muscle wasting additively in the setting of ageing. We investigated morphological characteristics in fast‐twitch extensor digitorum longus (EDL) muscle of mice fed a low‐fat diet (LFD) or a high‐fat diet (HFD) for 4 or 20 months. The fast‐twitch EDL muscle was harvested, and the muscle fibre‐type composition, individual muscle cross‐sectional area and myotube diameter were measured. We found an increase in the percentage of type IIa and IIx myosin heavy chain fibres in the whole EDL muscle, but a decrease in type IIB myosin heavy chain in both HFD protocols. The cross‐sectional area and myofibre diameter were lower in both groups of aged mice (after 20 months of LFD or HFD) compared with young mice (after 4 months of the diets), but there were no differences between mice fed LFD or HFD for 20 months. These data suggest that long‐term feeding of HFD does not aggravate muscle wasting in fast‐twitch EDL muscle of male mice.

## Abstract (highlights)

- **What is the central question of this study?**
  Ageing leads to a loss of mass in skeletal muscle, but the effect of obesity on ageing‐related muscle wasting is unclear. In this study, we aimed to demonstrate the specific effect of obesity on fast‐twitch skeletal muscle in ageing.
- **What is the main finding and its importance?**
  Our findings show that the obesity induced by long‐term ingestion of a high‐fat diet does not aggravate muscle wasting in fast‐twitch skeletal muscle of aged mice, indicating that the present study provides morphological characteristics for skeletal muscle of sarcopenic obesity.

## 1 INTRODUCTION

Age‐related loss of skeletal muscle mass and function is associated with physical frailty and increased risk of morbidity (Beard et al., 2016). In addition, obesity causes major health problems worldwide (Freedman et al., 2002), and the number of elderly obese individuals has increased dramatically (Zamboni & Mazzali, 2012). Studies have shown that maintenance of muscle mass is impaired in aged individuals (Bowen et al., 2015) and obese individuals (Akhmedov & Berdeaux, 2013), but the effect of obesity on ageing‐related muscle wasting is unclear.

It has been shown that rats (Tardif et al., 2014) or mice (L. A. Brown et al., 2021) fed a high‐fat diet (HFD) have reduced muscle mass in the tibialis anterior and reduced muscle cross‐sectional area (CSA) in gastrocnemius with ageing. Although skeletal muscle ageing has been shown primarily to affect fast‐twitch muscle (Nilwik et al., 2013), the gastrocnemius and tibialis anterior muscles are a mix of fast‐ and slow‐twitch muscle in rodents (Bloemberg & Quadrilatero, 2012). In contrast, Messa et al. (2020) showed that several weeks of HFD feeding does not change CSA in the fast‐twitch extensor digitorum longus (EDL) muscle in aged female mice. We previously demonstrated that long‐term HFD feeding additively impairs muscle contractile force in the EDL muscle in aged male mice (Eshima et al., 2020), suggesting that the period of diet feeding might involve impairment of muscle homeostasis. However, the specific effect of long‐term of HFD feeding on fast‐twitch skeletal muscle in sarcopenia has not been clarified fully.

Based on this background, we investigated muscle morphology in fast‐twitch EDL muscle in aged mice after long‐term HFD feeding. We tested the hypothesis that long‐term HFD feeding would enhance muscle wasting of fast‐twitch dominant muscle in ageing.

## 2 MATERIALS AND METHODS

### 2.1 Ethical approval

All animal experiments in this study were approved by the Animal Experimental Committee of Juntendo University (institutional ethics committee approval no. 290145). All experimental animals were handled according to institutional guidelines for the care and use of laboratory animals and conformed to the regulations described in the editorial by Grundy (2015). This manuscript was prepared according to the Animal Research: Reporting of In Vivo Experiments guidelines (https://arriveguidelines.org).

### 2.2 Animals

Male C57BL/6 male mice (7 weeks of age) were obtained from Charles River Laboratory Supplies and housed at a constant temperature of 22°C−24°C and a humidity of 50%−60%, with a controlled light–dark cycle (12 h−12 h). During a 1‐week adaptation period, all mice were fed standard chow and water ad libitum. After an initial acclimation period, animals (2 months of age) were randomly assigned to the following groups: 4 months of low‐fat diet (LFD; *n* = 6), 4 months of HFD (*n* = 6), 20 months of LFD (*n* = 6) or 20 months of HFD (*n* = 5). The 23 animals investigated herein represent a subset of mice from a larger cohort in which data related to assessment of the morphology have not been reported (Eshima et al., 2020). The published data confirmed that long‐term feeding of HFD accretes increased body weight and abdominal visceral fat, fasting glucose, but not fasting insulin, and homeostatic model assessment for insulin resistance in aged mice. The HFD mice were fed a diet containing 20% protein, 60% fat and 20% carbohydrate (D12492; Research Diets, New Brunswick, NJ, USA), and the LFD mice were fed standard chow containing 22.6% protein, 5.6% fat and 53.8% carbohydrate [a pelleted Charles River Formula (CRF)‐1 diet; Charles River, Japan]. After 4 months (when the mice were 6 months old) or 20 months (when the mice were 22 months old) of the HFD or standard chow diet, mice were anaesthetized via a single intraperitoneal injection of sodium pentobarbital (50 mg/kg was used for young LFD mice and 70 mg/kg for both HFD and all older mice), and their EDL muscles were quickly harvested once a surgical level of anaesthesia was reached. After harvest of the muscle, the mice were killed by cervical dislocation under general anaesthesia.

### 2.3 Histology

Mouse EDL muscle fibres were examined in histological sections and analysed as described previously (Eshima et al., 2017). Briefly, serial 10‐μm‐thick sections were cut with a cryostat (CM1510; Leica, Tokyo, Japan) at −20°C and mounted on polylysine‐coated slides. Whole sections were stained with Haematoxylin and Eosin or by immunohistochemistry for three fast myosin heavy chains (MyHCs). Mouse monoclonal antibodies that react specifically with type IIa (SC‐71) or type IIx (BF‐35) MyHC isoforms were supplied by the Developmental Studies Hybridoma Bank (University of Iowa, IA, USA). The M.O.M. Immunodetection kit (Vector Laboratories, Burlingame, CA, USA) and Vectastain ABC kit (Vector Laboratories) were used to assess immunohistochemical reactions according to the manufacturer's instructions. Images were recorded using a camera (E1000M; Nikon, Japan) at ×10 magnification and analysed subsequently in ImageJ (US National Institutes of Health, Bethesda, MD, USA). The CSA and Feret's diameter were measured by tracing fibre outlines of ∼371 fibres from the muscle sections of any individual mouse.

### 2.4 Statistical analysis

Statistical analyses were performed with Prism v.9.0 (GraphPad Software, San Diego, CA, USA). Data are presented as individual data points with the mean ± SD. For comparisons of groups, two‐way ANOVA with Holm–Sidak post hoc test was used to compare LFD and HFD groups. For all tests, *P* < 0.05 was considered statistically significant.

## 3 RESULTS

### 3.1 Fibre type

**FIGURE 1:**
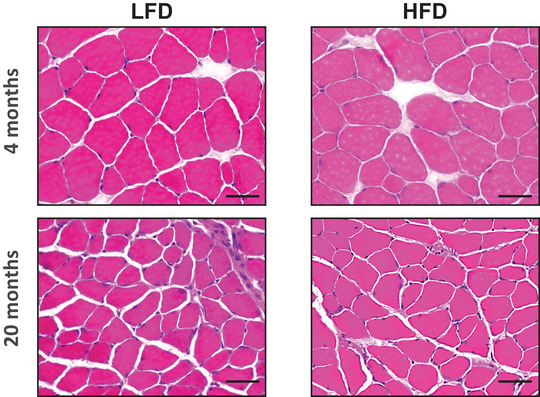
Transverse extensor digitorum longus muscle sections, stained with Haematoxylin and Eosin, from mice fed low‐fat diet (LFD) or high‐fat diet (HFD) for 4 or 20 months. Scale bars: 50 μm.

**FIGURE 2:**
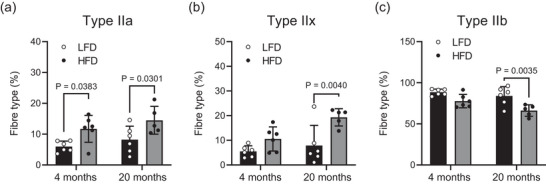
Fibre‐type composition for extensor digitorum longus muscle. (a) Myosin heavy chain (MyHC) type 2a. (b) MyHC type 2x. (c) MyHC type 2b. The presented data are the means ± SD [*n* = 6, 4 months of low‐fat diet (LFD); *n* = 6, 4 months of high‐fat diet (HFD) *n* = 6, 20 months of LFD; *n* = 5, 20 months of HFD].

As shown in Figure 1, mice fed LFD or HFD for 20 months demonstrated a decrease in individual EDL muscle CSA compared with the groups fed for 4 months, with no effects of diet. In addition, a shift in muscle fibre type from MyHC type IIb to type IIa/x was found in HFD groups, whereas no difference was observed in the shifts in fibre type between 4 and 20 months of HFD (Figure 2).

### 3.2 Cross‐sectional area and Feret's diameter

**FIGURE 3:**
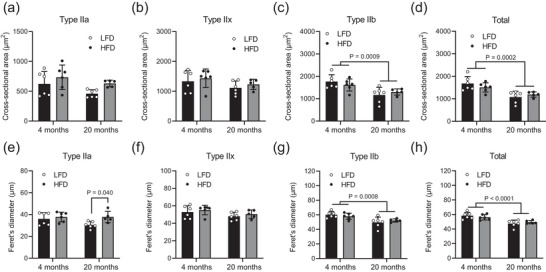
Muscle fibre cross‐sectional area and Feret's diameter by fibre type for extensor digitorum longus muscle. (a,e) Myosin heavy chain (MyHC) type 2a. (b,f) MyHC type 2x. (c,g) MyHC type 2b. (d,h) Total fibres analysed. The presented data are the means ± SD [*n* = 6, 4 months of low‐fat diet (LFD); *n* = 6, 4 months of high‐fat diet (HFD); *n* = 6, 20 months of LFD; *n* = 5, 20 months of HFD]. A main effect of ageing is shown in (c), (d), (g) and (h).

In MyHC type IIa/x from the 20 month groups, there was a trend of decreased CSA compared with those from 4 month groups (Figure 3a,b). For the MyHC type IIb and total analyses, CSA decreased significantly in the 20 month groups compared with the 4 month groups (Figure 3c,d). Ferret's diameter gave similar results (Figure 3e–h). However, the groups fed LFD and HFD for 20 months were not different regarding CSA and Feret's diameter.

### 3.3 Histograms of muscle fibre distribution

**FIGURE 4:**
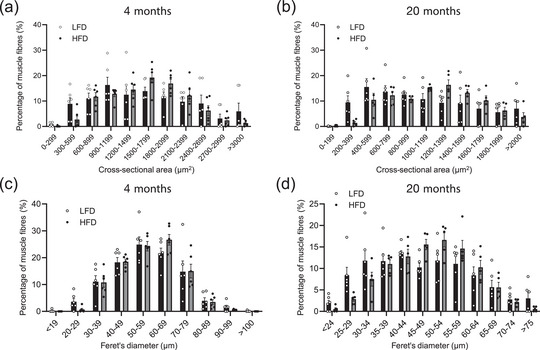
Distribution of muscle fibre cross‐sectional area and Feret's diameter for EDL muscle. (a) Cross‐sectional area for mice on low‐fat diet (LFD) or high‐fat diet (HFD) for 4 months. (b) Cross‐sectional area for mice on LFD or HFD for 20 months. (c) Feret's diameter for mice on LFD or HFD for 4 months. (d) Feret's diameter mice on LFD or HFD for 20 months. The presented data are the means ± SD (*n* = 6, 4 months of LFD; *n* = 6, 4 months of HFD; *n* = 6, 20 months of LFD; *n* = 5, 20 months of HFD).

Figure 4 shows histograms of CSA and Feret's diameter for each muscle fibre distribution. No significant differences according to diet were observed in CSA and Feret's diameter for either 4 months or 20 months. These data suggest that chronic HFD‐fed aged mice have unchanged muscle morphology in fast‐twitch dominant muscle.

## 4 DISCUSSION

We investigated the effect of chronic HFD feeding on muscle morphology of fast‐twitch EDL muscle of aged mice. Earlier studies have shown that chronic HFD feeding accelerates muscle atrophy in aged mice (L. A. Brown et al., 2021; Tardif et al., 2014). Interestingly, our data showed that the muscle morphology of aged obese mice was similar compared with ageing alone. In addition, HFD feeding did not change the CSA or Feret's diameter in the fast‐twitch EDL muscle after both 4 and 20 months. These findings suggest that chronic HFD feeding does not affect skeletal muscle atrophy in the fast‐twitch EDL muscle of aged mice.

Many studies have demonstrated that fast‐twitch muscle mass in mice is decreases with age (M. Brown & Hasser, 1996; Nilwik et al., 2013; Wohlwend et al., 2021). Regarding contractile function, we previously showed that HFD and ageing additively impair muscle contractile force in the EDL, but not the soleus muscle (Eshima et al., 2020). In addition, HFD impaired contractile force in the EDL together with changes in muscle fibre‐type composition (Eshima et al., 2017), suggesting that HFD alters the function of fast‐twitch fibres rather than slow‐twitch fibres. Regarding muscle mass in obese mice, previous studies showed that HFD‐induced obesity promotes muscle wasting, such as weakness, loss of muscle mass and decreased fibre diameter (Abrigo et al., 2016; Sishi et al., 2011; Sousa et al., 2021). In terms of the muscle mass affected by combined ageing and HFD, a recent study showed that long‐term feeding of HFD also decreased muscle CSA in the gastrocnemius of aged mice (L. A. Brown et al., 2021). However, the present study demonstrated that a chronic HFD did not exacerbate muscle atrophy in fast‐twitch EDL muscle during ageing. A previous study demonstrated that HFD‐induced obesity affects muscle mass of gastrocnemius muscle less in ageing (Lee et al., 2015). Consistent with these data, the present study demonstrated that morphological changes in fast‐twitch EDL muscle were less affected by a combination of ageing and HFD‐induced obesity. Indeed, the HFD did not affect the frequency distribution of CSA and Feret's diameter in individual fibres after 4 and 20 months of HFD (Figure 4a–d).

Previous reports show that ageing causes shifts in muscle fibre type in several hindlimb muscles (Faitg et al., 2019; Graber et al., 2015; Shang et al., 2020). However, we did not observe any differences in shifts of fibre type in fast‐twitch EDL muscle of aged mice. Consistent with a previous report (Eshima et al., 2017), the HFD‐fed groups showed an increased percentage of MyHC type IIa or IIx fibres, mainly at the expense of decreased type IIb fibres, compared with those from the LFD groups (Figure 2), suggesting that the dietary composition caused a shift in fibre type in skeletal muscle during ageing. In contrast, a previous report has demonstrated that short‐term (a few weeks) of HFD does not change fibre‐type composition of EDL in both young and aged mice (Hill et al., 2020; Messa et al., 2020). It is expected that the long period of feeding might promote fibre‐type shifts in the fast twitch EDL muscle, but further investigations are required to clarify the mechanisms.

Skeletal muscle mass and CSA in males are different from those in females (Hill et al., 2020). It has also been demonstrated that metabolic insult affects the sexes specifically. A previous study showed that insulin resistance occurs rarely in females or exclusively in males (Franconi et al., 2008). Supporting this, a higher level of adiposity is required in women to elicit metabolic disturbances than in males (Wajchenberg, 2000). Our study protocol was first investigated in males, which reliably became obese in response to the long‐term HFD feeding. The present study used only male mice, hence our data might not be generalized to a female model.

In conclusion, chronic HFD feeding did not exacerbate the muscle atrophy attributable to ageing in the fast‐twitch EDL muscle. Consistent with a previous report (Messa et al., 2020), the present study provides evidence that long‐term feeding of a HFD does not affect muscle morphology in ageing skeletal muscle. Further studies should examine the mechanism of skeletal muscle wasting in obesity and ageing.

## AUTHOR CONTRIBUTIONS

Hiroaki Eshima, Saori Kakehi, Ryuzo Kawamori, Hirotaka Watada and Yoshifumi Tamura, conceived and designed research; Hiroaki Eshima, performed experiments; Tsutomu Tagawa and Hiroaki Eshima analyzed data; Tsutomu Tagawa and Hiroaki Eshima interpreted results of experiments; Tsutomu Tagawa Hiroaki Eshima Saori Kakehi, Ryuzo Kawamori, Hirotaka Watada, and Yoshifumi Tamura, prepared figures; Tsutomu Tagawa, Hiroaki Eshima, and Yoshifumi Tamura, drafted the manuscript; Hirotaka Watada, Hiroaki Eshima, Saori Kakehi, Ryuzo Kawamori, Hirotaka Watada, and Yoshifumi Tamura, approved final version of the manuscript.

## CONFLICT OF INTEREST

None declared.

## Supporting information


Statistical Summary Document

## Data Availability

The datasets generated during and/or analysed during the present study are available from the corresponding author on reasonable request.
