# Supplementary material for: A chronic high‐fat diet does not exacerbate muscle atrophy in fast‐twitch skeletal muscle of aged mice
Source: Exp Physiol. 2023 Apr 19;108(7):940–5. doi: 10.1113/EP091106 (PMC10988437; doi:10.1113/EP091106)
Supplement: Supplementary file 1 — Statistical Summary Document [file EPH-108-940-s001.docx]

**Manuscript Title:** A chronic high fat diet does not exacerbate muscle atrophy in fast-twitch skeletal muscle of aged mice

**Authors:** Tsutomu Tagawa, Hiroaki Eshima, Saori Kakehi, Ryuzo Kawamori, Hirotaka Watada, and Yoshifumi Tamura

**Animal model used, if applicable: Mouse.** Male C57BL/6J background. High Fat Diet fed in 6 month old or 22 month old.

**Underlying hypothesis:** we investigated muscle morphology in fast-twitch dominant muscle, extensor digitorum longus (EDL) muscle in the long-term of HFD feeding in aged mice. We tested the hypotheses that obesity will further decrease the muscle wasting of fast-twitch dominant muscle in aging.

**Definitions of ‘n’:**

Question 1: n = number of animals from which muscle fiber type was stained and observed.

Question 2: n = number of animals from which average of muscle cross sectional area and feret’s diameter.

Question 3: n = number of animals from which histograms of muscle cross sectional area and feret’s diameter.

**Statistical summary table:**

| Experimental question number* | Finding/ conclusion | Experimental location/ variable  e.g. muscle, neocortex or genotype | Mean value  (or other summary statistic) | SD | n val. | P** | Units | Data comparisons  e.g. WT vs KO | Statistical test | Any other variable  e.g. subjects’ age or sex | Figure/ table in which data are presented | Comments  e.g. observation |
| --- | --- | --- | --- | --- | --- | --- | --- | --- | --- | --- | --- | --- |
| 1 How shifts muscle fiber type? | MHC type IIb to type IIa/x were found in HFD groups | IIa | 5.9853/11.7275  8.2027/14.4917 | 1.6094/ 3.9836  3.9877/4.0393 | 6/6  6/5 | P = 0.0383  P = 0.0301 | % | LFD vs HFD in 4mo  LFD vs HFD in 20mo | 2-way ANOVA  Sidak’s Multi. comp. |  | 2A |  |
|  |  | IIx | 5.5110/7.8874  10.5564/19.3216 | 2.0686/7.4454  4.4714/3.1353 | 6/6  6/5 | P = 0.2143  P = 0.0040 | % | LFD vs HFD in 4mo  LFD vs HFD in 20mo | 2-way ANOVA  Sidak’s Multi. comp. |  | 2B |  |
|  |  | IIb | 88.5036/83.9097  77.7160/66.1865 | 3.4293/10.3101  7.4226/6.2150 | 6/6  6/5 | P = 0.0622  P = 0.0035 | % | LFD vs HFD in 4mo  LFD vs HFD in 20mo | 2-way ANOVA  Sidak’s Multi. comp. |  | 2C |  |
| 2 Average of muscle cross sectional area | No differences in 20mo LFD and 20mo HFD groups | IIa | 623.2694/459.3206  733.5219/630.0985 | 188.8113/59.7527  188.0284/49.8040 | 6/6  6/5 | P = 0.4142  P = 0.1640 | μm^2^ | LFD vs HFD in 4mo  LFD vs HFD in 20mo | 2-way ANOVA  Sidak’s Multi. comp. |  | 3A |  |
|  |  | IIx | 1334.7169/1114.9086  1434.6589/1228.2377 | 334.8474/208.6766  283.4968/151.6994 | 6/6  6/5 | P = 0.7968  P = 0.7673 | μm^2^ | LFD vs HFD in 4mo  LFD vs HFD in 20mo | 2-way ANOVA  Sidak’s Multi. comp. |  | 3B |  |
|  |  | IIb | 1766.7771/1165.2809  1613.2345/1299.4530 | 277.6231/313.1211  244.5434/122.9125 | 6/6  6/5 | P = 0.5819  P = 0.6840 | μm^2^ | LFD vs HFD in 4mo  LFD vs HFD in 20mo | 2-way ANOVA  Sidak’s Multi. comp. | P = 0.0009  Main effect of age 4mo vs 20mo groups | 3C |  |
|  |  | Total | 1681.5718/1077.4904  1506.8935/1190.6908 | 284.2429/235.9584  187.9153/105.4588 | 6/6  6/5 | P = 0.4421  P = 0.8879 | μm^2^ | LFD vs HFD in 4mo  LFD vs HFD in 20mo | 2-way ANOVA  Sidak’s Multi. comp. | P = 0.0002  Main effect of age 4mo vs 20mo groups | 3D |  |
| 2 Average of feret’s diameter | No differences in 20mo LFD and 20mo HFD groups | IIa | 36.0379/30.5316  37.7477/37.7350 | 5.2018/2.8461  4.0443/4.6421 | 6/6  6/5 | P = 0.7835  P = 0.0396 | μm | LFD vs HFD in 4mo  LFD vs HFD in 20mo | 2-way ANOVA  Sidak’s Multi. comp |  | 3E |  |
|  |  | IIx | 52.8698/48.0808  55.1436/50.4615 | 6.6259/4.1955  4.9070/4.2871 | 6/6  6/5 | P = 0.7008  P = 0.6291 | μm | LFD vs HFD in 4mo  LFD vs HFD in 20mo | 2-way ANOVA  Sidak’s Multi. comp |  | 3F |  |
|  |  | IIb | 60.1737/49.5585  57.9890/52.1839 | 3.8797/6.6492  3.8267/2.0046 | 6/6  6/5 | P =0.7008  P = 0.6291 | μm | LFD vs HFD in 4mo  LFD vs HFD in 20mo | 2-way ANOVA  Sidak’s Multi. comp | P = 0.0008  Main effect of age 4mo vs 20mo groups | 3G |  |
|  |  | Total | 58.4105/47.5607 |  | 6/6  6/5 | P = 0.5930  P = 0.6809 | μm | LFD vs HFD in 4mo  LFD vs HFD in 20mo | 2-way ANOVA  Sidak’s Multi. Comp  2-way ANOVA  Sidak’s Multi. comp | P < 0.0001  Main effect of age 4mo vs 20mo groups | 3H |  |
| 3. histograms of muscle cross sectional area | No differences in 4mo LFD and 4mo HFD groups | \| 300-599 \| \| --- \| \| 600-899 \| \| 900-1199 \| \| 1200-1499 \| \| 1500-1799 \| \| 1800-2099 \| \| 2100-2399  2400-2699  2700-2999  >3000 \| \|  \| \|  \| \|  \| | \| \| 0.88/0.09 \| \| --- \| \| 8.89/2.72 \| \| 11.02/11.69 \| \| 16.29/12.82 \| \| 12.49/14.52 \| \| 13.89/19.22 \| \| 11.75/16.88 \| \| 9.72/12.14 \| \| 9.04/6.24 \| \| 3.13/2.37 \| \| 5.95/1.32 \| \| \| --- \| --- \| --- \| --- \| --- \| --- \| --- \| --- \| --- \| --- \| --- \| --- \| | \| 0.72/0.20  5.38/3.09  4.70/3.56  6.88/2.43  9.04/4.30  3.50/4.74  4.10/3.73  4.42/5.17  7.40/4.36  3.86/2.08  7.27/1.03 \| \| --- \| \|  \| | 6/6 | \| >0.9999 \| >0.9999 \| \| --- \| --- \| \| 0.3629 \| 0.3629 \| \| >0.9999 \| >0.9999 \| \| 0.9551 \| 0.9551 \| \| 0.9995 \| 0.9995 \| \| 0.5795 \| 0.5795 \| \| 0.6328 \| 0.6328 \| \| 0.9974 \| 0.9974 \| \| 0.9910 \|  \| \| >0.9999 \|  \| \| 0.7598 \|  \| | % | \| LFD vs HFD in 4mo \| \| --- \| \|  \| \|  \| \|  \| \|  \| \|  \| \|  \| \|  \| \|  \| | 2-way ANOVA  Sidak’s Multi. Comp |  | 4A |  |
| 3. histograms of muscle cross sectional area | No differences in 20mo LFD and 20mo HFD groups | \| 0-199 \|  \| \| --- \| --- \| \| 200-399 \|  \| \| 400-599 \|  \| \| 600-799 \|  \| \| 800-999 \|  \| \| 1000-1199 \|  \| \| 1200-1399 \|  \| \| 1400-1599 \|  \| \| 1600-1799 \|  \| \| 1800-1999 \|  \| \| >2000 \|  \| | \| 0.04/0.22 \| \| --- \| \| 9.43/1.41 \| \| 15.54/10.48 \| \| 13.71/12.25 \| \| 12.48/10.84 \| \| 10.75/14.95 \| \| 9.31/16.35 \| \| 9.14/13.22 \| \| 6.99/10.23 \| \| 5.59/6.29 \| \| 7.01/3.75 \| | \| \| 0.10/0.27 \| \| --- \| \| 5.93/1.08 \| \| 7.28/5.08 \| \| 5.19/3.19 \| \| 3.48/0.92 \| \| 4.91/0.97 \| \| 3.90/4.01 \| \| 7.13/2.67 \| \| 5.21/3.59 \| \| 3.87/2.55 \| \| 5.90/3.39 \| \| \| --- \| --- \| --- \| --- \| --- \| --- \| --- \| --- \| --- \| --- \| --- \| --- \| | 6/5 | \| >0.9999  0.0678  0.6081  >0.9999  >0.9999  0.8271  0.1641  0.8514  0.9650  >0.9999  0.9633 \| \| --- \| | % | LFD vs HFD in 20mo | 2-way ANOVA  Sidak’s Multi. Comp |  | 4B |  |
| 3. histograms of muscle feret’s diameter | No differences in 4mo LFD and 4mo HFD groups | \| <19 \| \| --- \| \| 20-29 \| \| 30-39 \| \| 40-49 \| \| 50-59 \| \| 60-69 \| \| 70-79 \| \| 80-89 \| \| 90-99 \| \| >100 \| | \| 0.18/0.00 \| \| --- \| \| 3.62/0.71 \| \| 11.05/10.78 \| \| 18.28/18.46 \| \| 24.87/24.27 \| \| 21.60/26.67 \| \| 14.77/15.01 \| \| 3.94/3.49 \| \| 1.59/0.61 \| \| 0.09/0.00 \| | \| \| 0.41/0.00 \| \| --- \| \| 2.71/0.93 \| \| 5.02/3.97 \| \| 4.02/2.21 \| \| 6.39/3.96 \| \| 4.40/4.43 \| \| 8.99/5.71 \| \| 2.61/2.03 \| \| 1.59/0.15 \| \| 0.21/0.00 \| \| \| --- \| --- \| --- \| --- \| --- \| --- \| --- \| --- \| --- \| --- \| --- \| | \| 6/6 \| \| --- \| \|  \| \|  \| \|  \| \|  \| \|  \| \|  \| \|  \| \|  \| \|  \| | >0.9999  0.9282  >0.9999  >0.9999  >0.9999  0.3261  >0.9999  >0.9999  >0.9999  >0.9999 | % | LFD vs HFD in 4mo | 2-way ANOVA  Sidak’s Multi. Comp |  | 4C |  |
| 3. histograms of muscle feret’s diameter | No differences in 20mo LFD and 20mo HFD groups | \| <24 \| \| --- \| \| 25-29 \| \| 30-34 \| \| 35-39 \| \| 40-44 \| \| 45-49 \| \| 50-54 \| \| 55-59 \| \| 60-64 \| \| 65-69 \| \| 70-74 \| \| >75 \| | \| 1.87/0.54 \| \| --- \| \| 8.41/2.84 \| \| 11.81/7.49 \| \| 11.70/11.02 \| \| 13.23/12.77 \| \| 10.24/15.56 \| \| 11.82/16.63 \| \| 11.07/14.63 \| \| 8.38/10.26 \| \| 5.63/5.59 \| \| 2.79/2.11 \| \| 3.04/0.55 \| | \| \| 1.54/0.61 \| \| --- \| \| 4.12/1.06 \| \| 5.72/3.24 \| \| 3.83/1.99 \| \| 2.02/3.38 \| \| 2.16/2.40 \| \| 4.00/3.30 \| \| 4.38/3.80 \| \| 4.44/3.23 \| \| 3.57/2.36 \| \| 1.76/0.65 \| \| 3.04/0.50 \| \| \| --- \| --- \| --- \| --- \| --- \| --- \| --- \| --- \| --- \| --- \| --- \| --- \| --- \| |  | \| 0.9999  0.1087  0.4107  >0.9999  >0.9999  0.1461  0.2569  0.6954  0.9964  >0.9999  >0.9999  0.9625 \| \| --- \| | % | LFD vs HFD in 20mo | 2-way ANOVA  Sidak’s Multi. Comp |  | 4D |  |

*You may use multiple lines for the same question to indicate multiple comparisons

** Authors may wish to make the text bold where p is considered significant against a stated confidence limit.
